# Direct Determination of Torsion in Twisted Graphite and MoS_2_ Interfaces

**DOI:** 10.1021/acs.nanolett.4c01944

**Published:** 2024-07-11

**Authors:** Gautham Vijayan, Elad Koren

**Affiliations:** Nanoscale Electronic Materials and Devices Laboratory, Faculty of Materials Science and Engineering, Technion - Israel Institute of Technology, Haifa 3200003, Israel

**Keywords:** 2D materials, graphite, MoS_2_, torsion, mechanical actuation

## Abstract

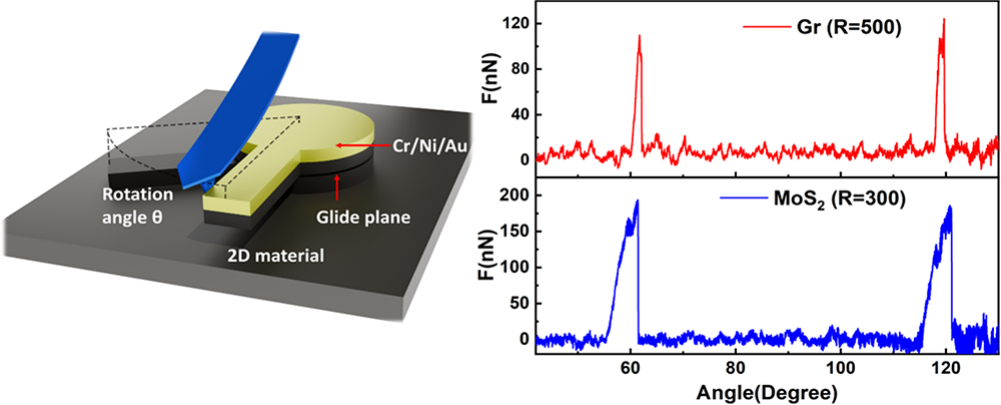

The design space of two-dimensional materials is undergoing significant expansion through the stacking of layers in non-equilibrium configurations. However, the lack of quantitative insights into twist dynamics impedes the development of such heterostructures. Herein, we utilize the lateral force sensitivity of an atomic force microscope cantilever and specially designed rotational bearing structures to measure the torque in graphite and MoS_2_ interfaces. While the extracted torsional energies are virtually zero across all angular misfit configurations, commensurate interfaces of graphite and MoS_2_ are characterized by values of 0.1533 and 0.6384 N-m/m^2^, respectively. Furthermore, we measured the adhesion energies of graphite and MoS_2_ to elucidate the interplay between twist and slide. The adhesion energy dominates over the torsional energy for the graphitic interface, suggesting a tendency to twist prior to superlubric sliding. Conversely, MoS_2_ displays an increased torsional energy exceeding its adhesion energy. Consequently, our findings demonstrate a fundamental disparity between the sliding-to-twisting dynamics at MoS_2_ and graphite interfaces.

Two-dimensional (2D) materials enable stacking of individual layers in non-equilibrium configurations, for instance, via adjustment of the twist angle, significantly extending the design space for heterostructures. The relative twist angle between layers modifies the crystal structure, leading to a variety of intriguing physical properties such as superconductivity,^1,2^ ferroelectricity,^3−5^ moiré excitons,^6,7^ and structural superlubricity.^8−10^ Currently, researchers primarily utilize the angular degree of freedom to manipulate material characteristics by the intentional alignment of individual constituents using static alignment techniques such as dry transfer,^11,12^ water-assisted transfer,^13^ thermal annealing,^14,15^ and colamination mechanical transfer.^16^ However, there is no available information regarding the torque required to create a dynamic misalignment from an equilibrium state and its size dependence, which is essential for precise control and manipulation.

In addition, the interplay between twisting and sliding plays a crucial role in the mechanical alignment and actuation of 2D materials as well as their assembly into functional devices using layer-by-layer techniques. For instance, structural superlubricity arises from lattice incommensurability between sliding surfaces, which leads to vanishing friction at the interface.^17−19^ Furthermore, experimental and theoretical studies of incommensurate layers showed that the layers often undergo self-realignment to a locking position followed by a stick–slip movement.^9^ The abrupt jump to a commensurate configuration is facilitated by the torque generated at the interface, which results in an increased friction. The torsional realignment to commensurate positions in 2D materials such as graphite occurs only at angular intervals of 60°, owing to the symmetry of the hexagonal lattice. In other cases, it was reported that prior to sliding, the interface underwent rotation to a noncommensurate configuration.^20^ Therefore, quantifying torque and the interplay between sliding and twisting in 2D materials is crucial for comprehensive understanding of adhesion, friction, directional manipulation, and non-equilibrium stacking.

Herein, we employ the lateral deflection of an atomic force microscopy (AFM) cantilever to quantitatively measure torque in graphite and MoS_2_ interfaces. In particular, we utilize a unique experimental technique that allows rotation of the interface, while keeping the center of rotation and contact area intact by the stabilizing adhesive line tension forces. Discrete torque peaks were observed at commensurate configurations with an angular interval of 60°, attributed to the inherent symmetry of the crystal lattices, whereas no measurable torque was observed across incommensurate configurations. In addition, torque at commensurate configurations demonstrates a linear dependence as a function of the interfacial area in both circular graphite and MoS_2_ mesostructures. Furthermore, we measured the adhesion for MoS_2_ and graphite mesostructures to address the interplay between sliding and rotational energy barriers. The adhesion energy of the graphitic interface was found to be higher than its torsional energy, explaining the greater susceptibility of a commensurate interface to twist prior to sliding. On the contrary, the torsional energy of MoS_2_ is higher than its adhesion energy. Thus, our study indicates a fundamental difference in the interplay between sliding and twisting in MoS_2_ and graphite, with a potential impact on directional manipulation and structural assembly of 2D layered materials.

High-quality HOPG (MikroMasch, ZYA grade) was utilized as the substrate for graphite, and a MoS_2_ film (Manchester Nanomaterials) with a thickness of ∼120 nm was mechanically exfoliated onto a Au/Cr/SiO_2_/Si wafer, serving as the support substrate. Electron beam lithography was used to fabricate cylindrically shaped metal contacts (5 nm Cr, 25 nm Ni, and 25 nm Au) of different radii (200, 250, 300, and 500 nm) equipped with a lever arm for rotational manipulation. Finally, reactive ion etching was used to construct the pillar structures with an average height of 80 nm, where metal contacts served as the etch mask (a detailed description of the fabrication process is provided in section 1 of the Supporting Information).

The experiments were performed using an AFM instrument (Bruker Dimension V) situated inside a nitrogen-filled glovebox (H_2_O and O_2_ content of <1 ppm). Schematic representation of the experimental setup is depicted in Figure 1a. In particular, a Pt/Ir-coated AFM tip (Nanosensors-PPP-NCLPt) was precisely positioned next to the lever arm at a radial distance of ∼1 μm from the center of the circular structure, and a circular motion was executed along a defined angle with the pillar serving as the center of rotation. The mechanical actuation allows breaking of the pillar at a single glide plane, where the top section is continuously rotating over the fixed bottom section. Despite the noncentric actuation, the rotation axis remains located at the center of the pillar due to stabilizing adhesive line tension forces.^21,22^Figure 1b demonstrates a typical height profile of a 180°-rotated MoS_2_ structure with an inset showing the cross-sectional AFM topography. Lateral deflection and *Y*-axis displacement of the cantilever circular trajectory were recorded using an oscilloscope (Keysight DSOS054A) throughout the rotation and were used to obtain the force angle profiles. The reported adhesion energy of MoS_2_ was utilized to calibrate the lateral force constant of the AFM cantilever^23^ (section 2 of the Supporting Information). The piezo displacement along the *Y*-axis was subsequently converted into angular displacement^22^ (section 3 of the Supporting Information). Figure 1c illustrates typical force angle profiles, indicating two distinct peaks occurring at an interval of 60° for both graphite and MoS_2_, where the force peaks at 60° are roughly double the height of the force peaks at 120°. The asymmetry in the measured force for 60° and 120° can be explained on the basis of the schematic top view of the experiment (Figure 2a), where the initial contact with the pillar arm comprises 60° between the AFM probe and the *Y*-axis cantilever trajectory (position 1). Thus, the lateral deflection along the *Y*-axis perfectly aligns with the first commensurate configuration at 60° with respect to the circular tip trajectory (position 2), effectively capturing its entire force magnitude. Beyond position 2, the lateral deflection reflects only a fraction of the real force (*F*_real_) required to rotate the interface (see section 4 of the Supporting Information). Notably, this effect is evident in Figure 1c, wherein the measured force peak magnitude at 120° (position 3; cos 60 = ^1^/_2_) is approximately half of that of the initial peak at 60° (position 2; cos 0 = 1). This observation indicates that the measured force (*F*_meas_) follows a cosine modulation with respect to the angle of the *Y*-axis, facilitating the determination of the real force at the interface by utilizing the following relationship:
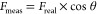
1It is important to note that eq 1 is valid for only the aforementioned sample orientation. Equation 1 was used to reconstruct the correct force angle profile (Figure 2b), where two identical force peaks with a separation of 60° were confirmed for both graphite and MoS_2_ structures. In contrast, no discernible force was detected between the commensurate positions. A comparable phenomenon has been observed in previous shearing experiments, particularly in graphite, wherein torsional realignment toward commensurate positions occurs at sliding angles that are multiples of 60°.^9,24^ Furthermore, distinct force barriers were detected in the hBN–graphene heterojunction at angular multiples of 60°.^25^ These observations imply that a significant torsional force is required solely at commensurate configurations, attributed to the inherent symmetry of the interface. Hence, our findings align with the 6-fold angular symmetry of torsion, which corresponds to the hexagonal lattice structures of both graphite and MoS_2_.

**Figure 1:**
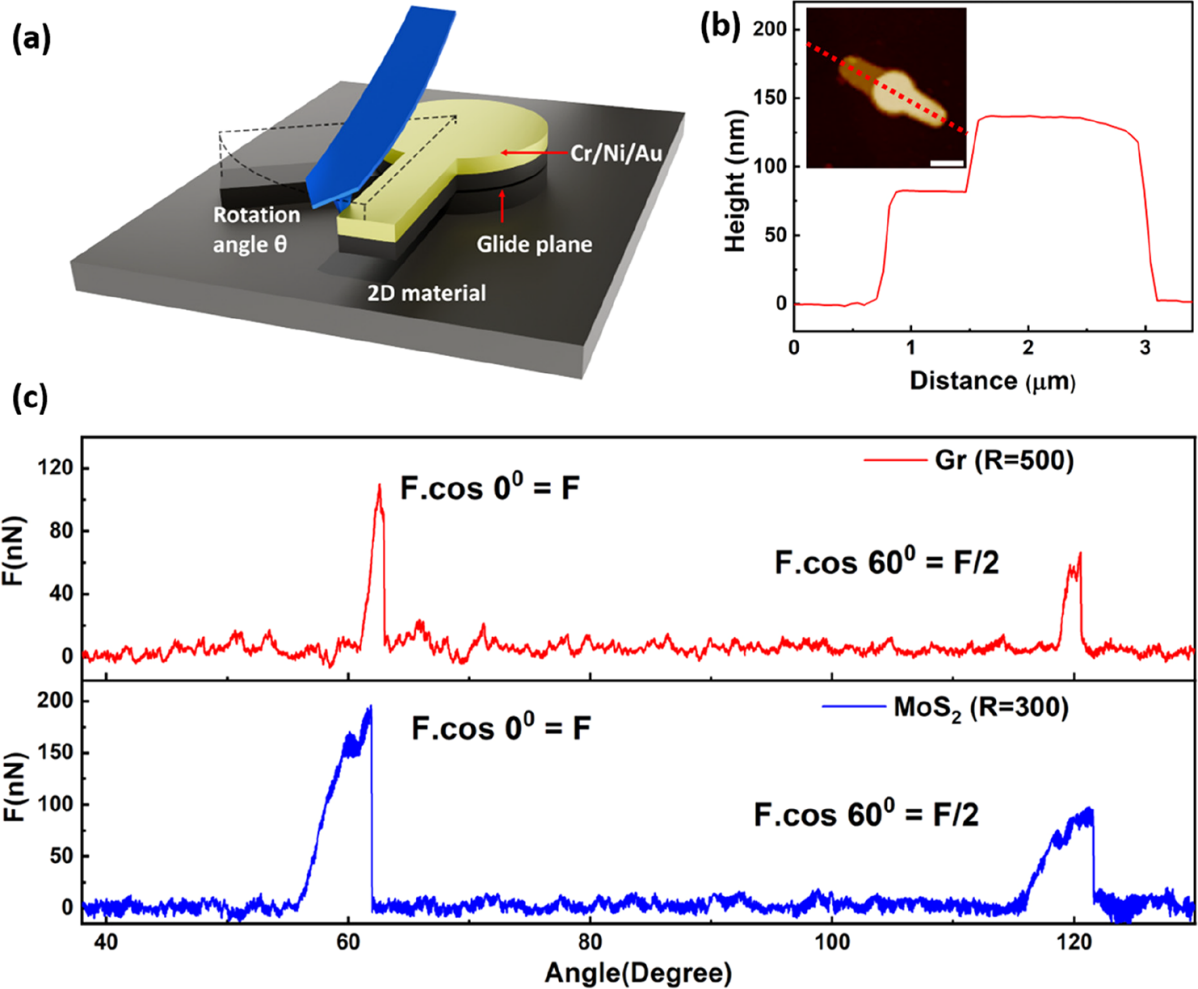
(a) Schematic illustration of the experimental setup for creating a single twisted interfacial defect within MoS_2_ or graphite pillars with precise control over the angular configuration while maintaing a constant area overalp. (b) Height profile of the MoS_2_ pillar rotated by 180°. The inset shows the AFM topography in which the dotted red line corresponds to the height profile. The scale bar is 600 nm. (c) Force vs angle profiles for graphite (red line, pillar radius of 500 nm) and MoS_2_ (blue line, pillar radius of 300 nm).

**Figure 2:**
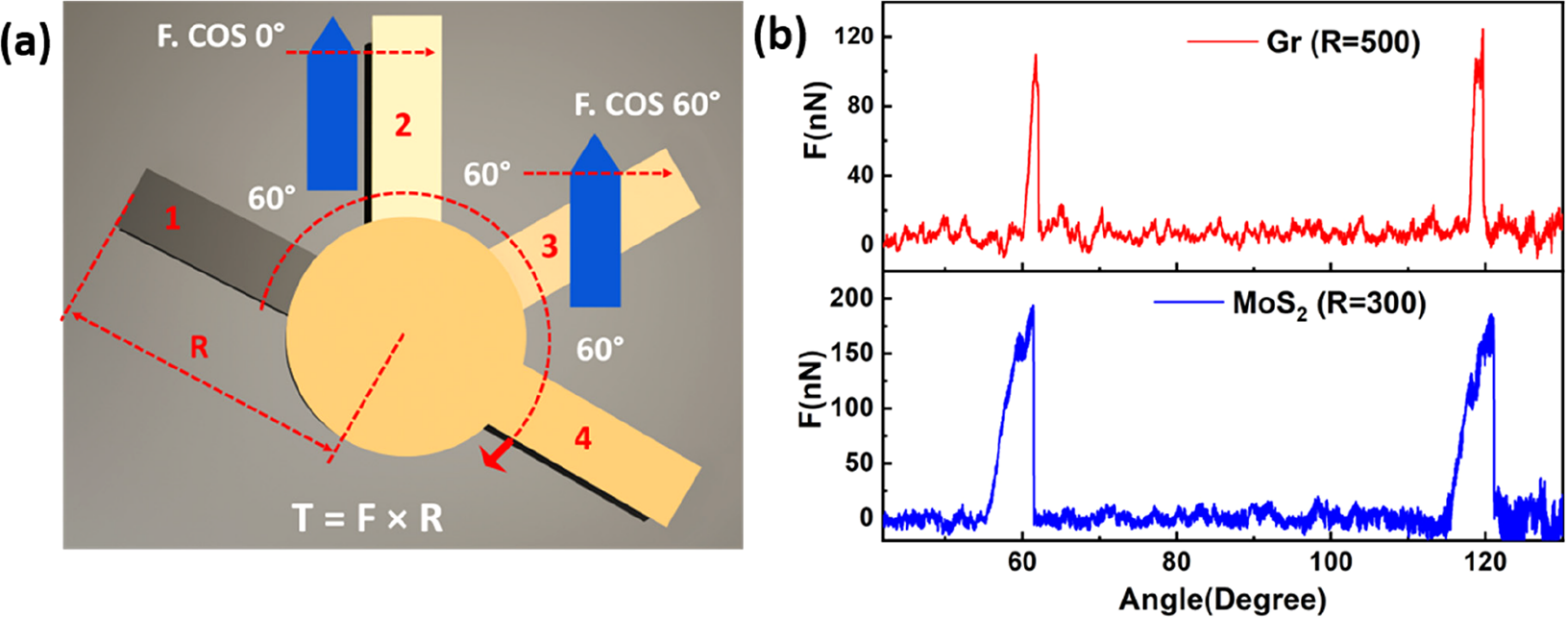
(a) Schematic illustration of the pillar orientation with respct to the commensurate positions and *Y*-axis of the sliding AFM cantilever. Commensurate positions are marked from 1 to 4. Cantilver contact points at positions 2 and 3 correspond to the measured force peaks in panel b. (b) Force vs angle profiles corrected according to eq 1 for graphite (red line, pillar radius of 500 nm) and MoS_2_ (blue line, pillar radius of 300 nm).

To investigate the possibility of additional commensurate configurations and further validate eq 1, further experiments were conducted by modifying the angular orientation of the sample to comprise a symmetric orientation of 30° between the *Y*-axis and two consecutive commensurate configurations as depicted in Figure 3a. Interestingly, the lever arm exerted an equal absolute amount of force on the cantilever at angular misfits of 60°, 120°, 240°, and 300° (Figure 3b). The symmetrical nature of cantilever interactions at angular intervals of 60° agrees with eq 1 and further confirms the virtually zero force barrier across noncommensurate misfit angles. The absence of force peaks at positions 1 and 4 is attributed to the parallel alignment of the lateral deflection axis with the lever arm. The AFM topography of locking configurations at angular intervals of 60° during mechanical actuation is shown in Figure 3c.

**Figure 3:**
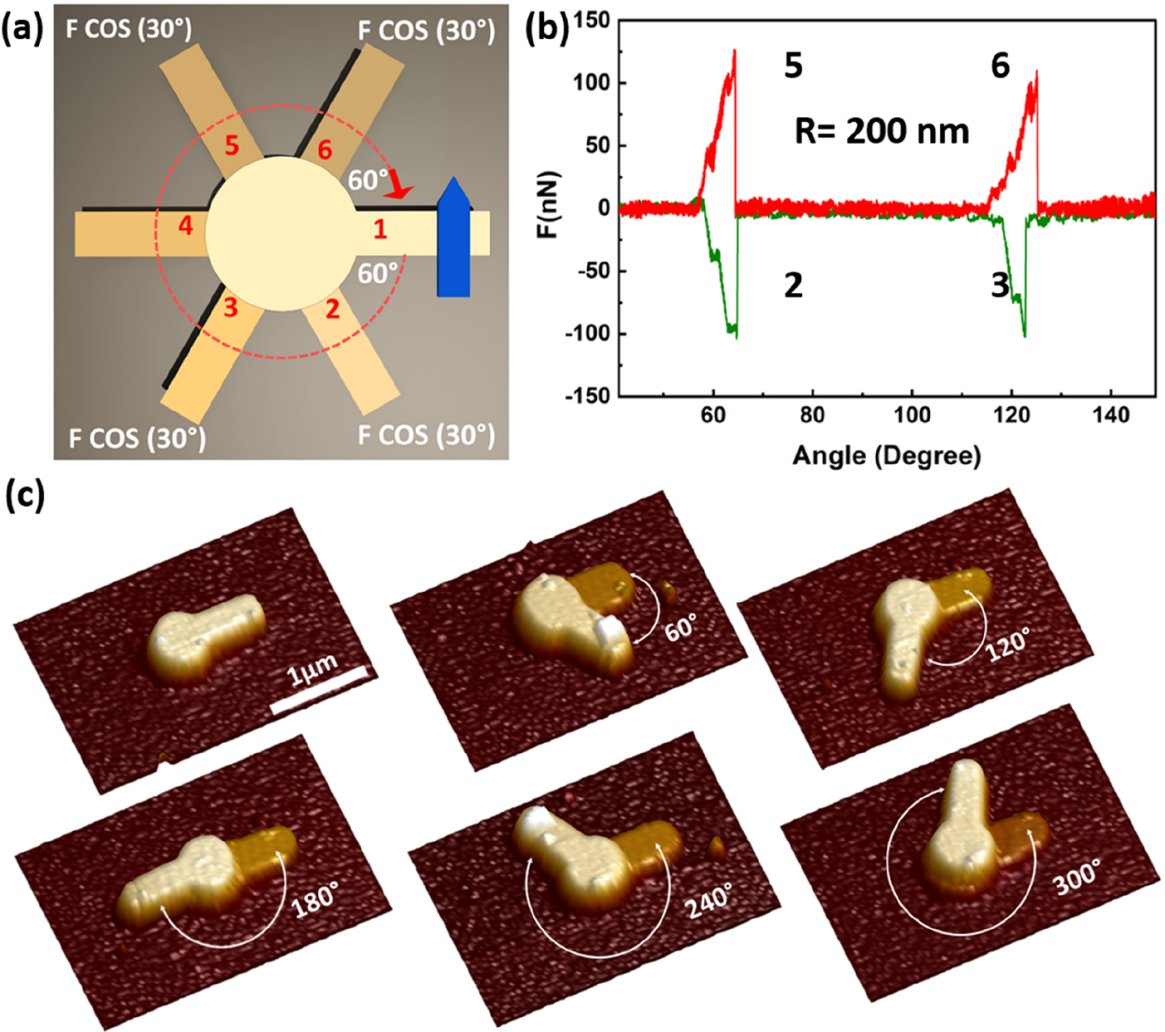
(a) Schematic illustration of the pillar rotation with commensurate positions marked from 1 to 6 for 360° rotation. (b) Force vs angle profiles for MoS_2_ during rotation. The green line corresponds to actuation from 0° to 180°, and the red line corresponds to the actuation from 180° to 360°. Commensurate positions are marked beside the force peak. (c) Three-dimensional AFM topography of the commensurate locking positions of 0°, 60°, 120°, 180°, 240°, and 300°.

Next, we studied the force scaling relative to the pillar areal size. The measured structures were aligned such that the commensurate configuration fits with the *Y*-axis to directly measure *F*_real_, similar to that shown in Figure 2. In particular, the torque was computed by multiplying the force magnitude at the commensurate configuration by the radius of the circular trajectory, as illustrated in Figure 2a. The torque per unit area (torsional energy) was defined by conducting torsional experiments for different pillar radii, as presented in Figure 4. The insets show the force angle profiles obtained for various radii of MoS_2_ and graphite at 60°. The linear regression slope results in normalized torques of 0.6384 N-m/m^2^ for MoS_2_ and 0.1533 N-m/m^2^ for graphite. It is evident that the torsional energy between MoS_2_ interfaces is ∼4 times higher that of graphite. At the same time, in numerous instances for both materials, achieving pillar-centric rotation was difficult, resulting in the displacement of the top mesa from the center of the bottom mesa. These observations suggest that the mechanism governing the movement between two distinct crystalline layers involves a dynamic interplay between sliding and rotation along energetically favorable pathways.

**Figure 4:**
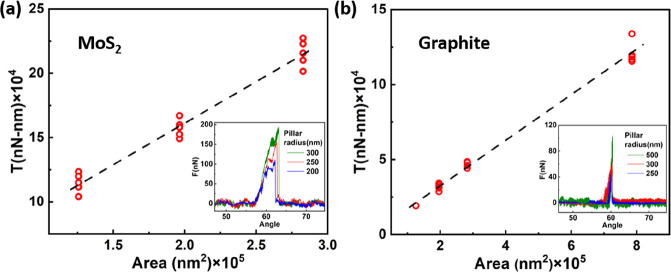
Torque at the commensurate configuration vs contact area for (a) MoS_2_ and (b) graphite. The dashed line corresponds to the linear fit. The insets present the force profile at angular misfits of 60° for different pillar radii.

Therefore, to gain a better understanding regarding the interplay between sliding and rotation, circular pillar structures composed of MoS_2_ and graphite were subjected to lateral shearing to quantify their adhesion energy (see section 2 of the Supporting Information). In particular, while the known adhesion value of MoS_2_ (i.e., 0.501 J/m^2^) was used for force calibration, an adhesion energy of 0.210 J/m^2^ was measured for graphite. Notably, the measured torsional energies denote the energy required to induce a twist without altering the interfacial area. Thus, in the case of graphite, the torsional energy is significantly lower than the adhesion energy. Hence, starting from a commensurate configuration, the graphite interface tends to undergo energetically favorable rotational motion rather than sliding when subjected to an external lateral force. This explains the intriguing phenomenon observed in previous studies of graphite,^20^ where the top layers consistently exhibit a twisted trajectory prior to laterally induced sliding actuation. The behavior of MoS_2_ is the opposite of that of graphite, favoring sliding over rotation once positioned at a commensurate configuration.

To further explore the interplay between sliding and rotation, it is essential to quantify the sliding forces in a scenario where the top layer slides over an infinite bottom layer in different angular mismatch configurations without altering the overlapping area. Conducting such experiments proves to be extremely challenging, and literature data are limited for both MoS_2_ and graphite. However, a similar experimental^26^ and theoretical^27^ study was conducted on graphite interfaces, involving the manipulation of graphite nanoflakes across an infinite graphite surface using a scanning tunneling microscope at ultralow temperatures. The observations indicate a consistent pattern in which the flake jumps initially from a commensurate state to an incommensurate state through rotational motion. Subsequently, the flake undergoes a combination of translational and rotational movements, continuing its propagation along several commensurate positions, driven by the initial kinetic energy. Our experiments demonstrate that rotational motion between 60° intervals requires minimal torque, enabling the seamless combination of rotational and translational motion subsequent to linear actuation as observed in the aforementioned reports. Nevertheless, conducting further precise experiments is imperative for gaining a more quantitative understanding of the actuation dynamics.

In summary, torque versus angular misfit configurations was determined for both MoS_2_ and graphite. The torque exhibited a 6-fold symmetry, consistent with the crystal structures and linear correlation with respect to the interfacial area. Furthermore, the adhesion energies of MoS_2_ and graphite interfaces were determined through shearing experiments and compared with the torsional energy. The adhesion energy of the graphite interface was found to be significantly higher than its torsional energy, suggesting that a commensurate configuration is more susceptible to twisting rather than sliding. Conversely, the torsional energy of MoS_2_ is slightly higher than its adhesion energy. Our experimental results provide quantitative insights into the interplay between sliding and rotation in 2D material interfaces, offering valuable information for the development of dynamic heterostructures for diverse electromechanical applications and assembly of devices.

## Supporting Information Available

Fabrication of circular mesa structures, tip calibration and adhesion measurements, angle calculation, and torque calculation (PDF)
