# Supplementary material for: Direct Determination of Torsion in Twisted Graphite and MoS2 Interfaces
Source: Nano Lett. 2024 Jul 11;24(29):8973–8. doi: 10.1021/acs.nanolett.4c01944 (PMC11273615; doi:10.1021/acs.nanolett.4c01944)
Supplement: Supplementary file 1 — nl4c01944_si_001.pdf [file nl4c01944_si_001.pdf]

Direct Determination of Torsion in Twisted Graphite and MoS_2 Interfaces

Gautham Vijayan and Elad Koren*

Nanoscale Electronic Materials and Devices Laboratory, Faculty of Materials Science and Engineering, Technion - Israel Institute of Technology, Haifa, 3200003, Israel.

*Email – eladk@technion.ac.il

Supplementary Information

1. Sample Fabrication

Both substrates (Bulk HOPG and Exfoliated MoS_2 on Si wafer) were spin coated with polymethyl methacrylate (PMMA) photoresist. Electron beam lithography (Raith EBPG 5200) was employed to define the pillar shape onto the photoresist coated substrates. A short oxygen plasma was used to clean the resist residuals prior to metal deposition. The metal deposition was carried out by electron beam evaporation (Evatec BAK-501A) – 5 nm of Cr were deposited to form good adhesion, followed by 25 nm Ni, and 25 nm Au. The photoresist was lifted off from the substrates using acetone, and the final pillar structures were formed using reactive ion etching (RIE- Plasma-Therm 790). Oxygen plasma was used to etch HOPG and MoS_2 was etched using SF_6 (200 W, 20 sccm and 20 mT). Detailed schematics of the aforementioned processes are described below.

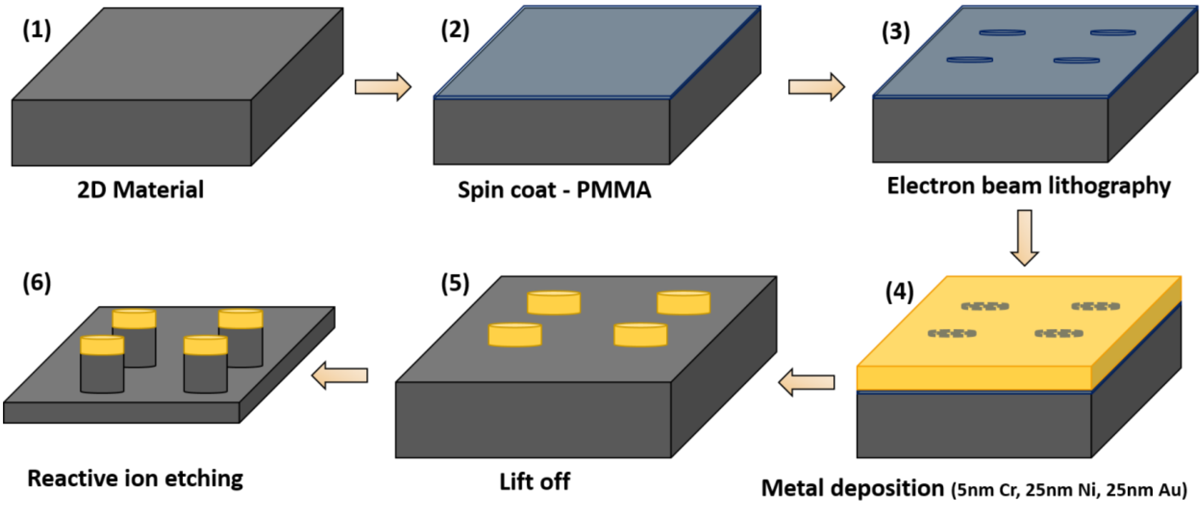

Figure S1. Schematics of the steps involved in fabrication proces. (1) Pristine 2D material(HOPG or MoS_2). (2) Spin coating is performed on the substrate to cover with PMMA as photoresist. (3) Electron beam lithography is employed to write circular as well as bearing pillar structures for nanomanipulation. (4)Metal depostion is performed on the entire substrate. (5) Lift off process results in metal pillars precisely at the patterened positions. (6) RIE process produces pillar structures of 2D materials with metal contacts serving as the etch mask.

2. Tip Calibration and Adhesion measurements

Single AFM tip (Nanosensors – PPP-NCLPt) was used for all the torque and adhesion measurements to eliminate statistical error due to cantilever variations. We use the adhesion energy of MoS_2 (σ = 0.501 J/m^2) from literature[1] to calibrate the lateral force constant of the AFM cantilever. For adhesion measurements, we sheared circular pillar structures of graphite and MoS_2 (Figure S2) separately and measured the corresponding lateral deflection of the cantilever in volts as depicted in Figure S3. As illustrated in the force - distance profiles, the lateral deflection initially increases upon the AFM tip's contact with the pillar structure, followed by a sudden drop at the onset of sliding. During the sliding process, new interfacial area is created which is opposed by the line tension forces arising from the interfacial energy. The line tension force acting along the slide direction can be defined as[2],

$$F = \sigma \frac{dA}{dx} \quad \text{(S1)}$$

where σ denotes the adhesion energy per unit area, A is the overlapping area between the mesa sections and x denotes the distance moved along the slide direction from the initial position. We know that for a circular mesa with a radius of r, instantaneous overlapping area (A), can be described as,

$$A(x) = 2r^2 \left[\cos^{-1}\left(\frac{x/2}{r}\right) - \frac{x/2}{r} \sqrt{1 - \left(\frac{x/2}{r}\right)^2} \right] \quad \text{(S2)}$$

Equation S1 can be rewritten by substituting for A from equation S2 as[2],

$$F(x) = -2\sigma r \sqrt{1 - \frac{(x/2)^2}{r^2}} \quad \text{(S3)}$$

The cantilever experiences maximum force during the shearing process when the initial displacement is zero. Hence, the maximum lateral force at the onset of shearing ($F_A(MoS_2)$ and $F_A(Gr)$) can be directly measured for a known radius (r) based on the following expression[2,3],

$$F_A = 2\sigma r \quad \text{(S4)}$$

And consequently, the lateral force constant c can be used to convert the measured lateral deflection from volts to Newton by substituting a known adhesion energy (for MoS_2, σ = 0.501 J/m^2) in the expression $F=2\sigma r=c\times V$. The obtained lateral force constant is employed to convert the lateral deflection from volts to nano Newton (nN) in further shearing and torsion experiments.

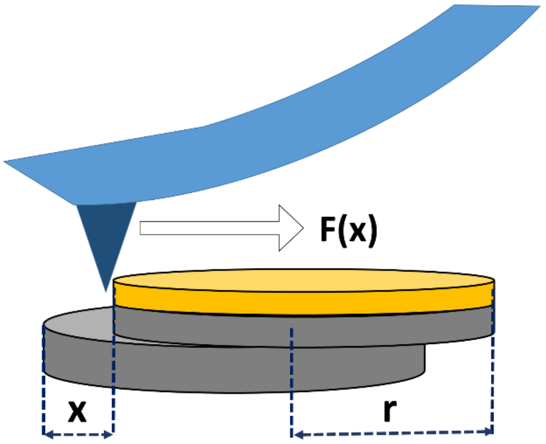

Figure S2. Schematic of the adhesion measurement. r denotes the pillar radius. The sliding distance from the initial position is defined as x and the corresponding sliding force experienced by the cantilever is F(x).

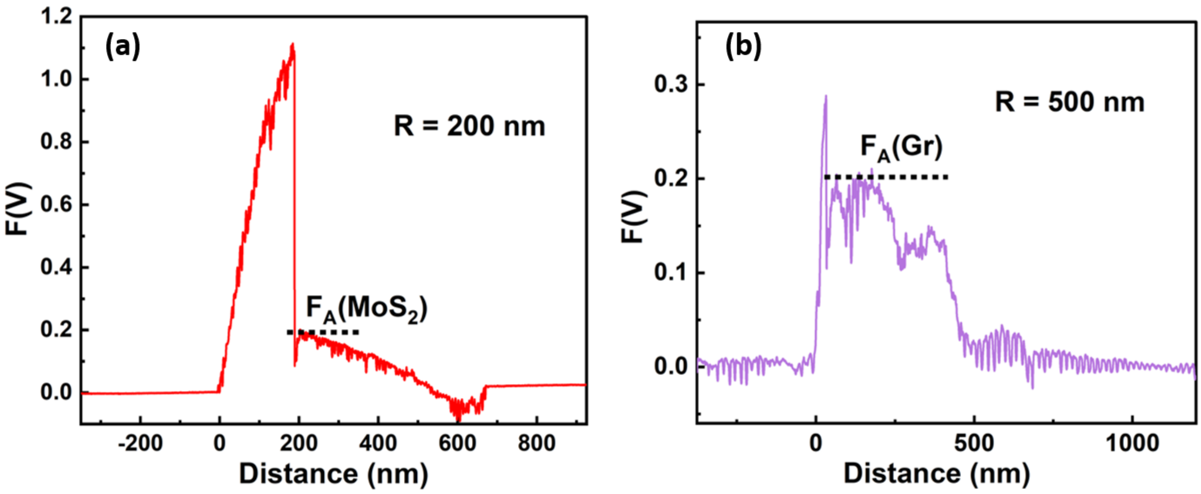

Figure S3. (a) Lateral force profile for MoS_2 mesa structure of radius 200 nm. (b) Lateral force profile for graphite mesa structure of radius 500 nm.

Finally, we note that although we do not have a direct way to verify the angular mismatch during the lateral sliding measurements, previous experimental report has shown that the adhesion does not depend strongly on the angular mismatch and is therefore mainly attributed to the new area exposure[4].

3. Angle calculation

During the experiment, we record the time trace of cantilever displacement along the Y axis and converted to angular units using the expression below.

$$\theta = t \times \frac{\Delta\theta}{\Delta t}$$

Δt is the time required to complete the actuation (Fig. S4). $\Delta\theta$ is the angle defined for the circular trajectory during mechanical actuation from point (x1,y1) to (x2,y2) with radius r_{scan} (inset in Fig. S4).

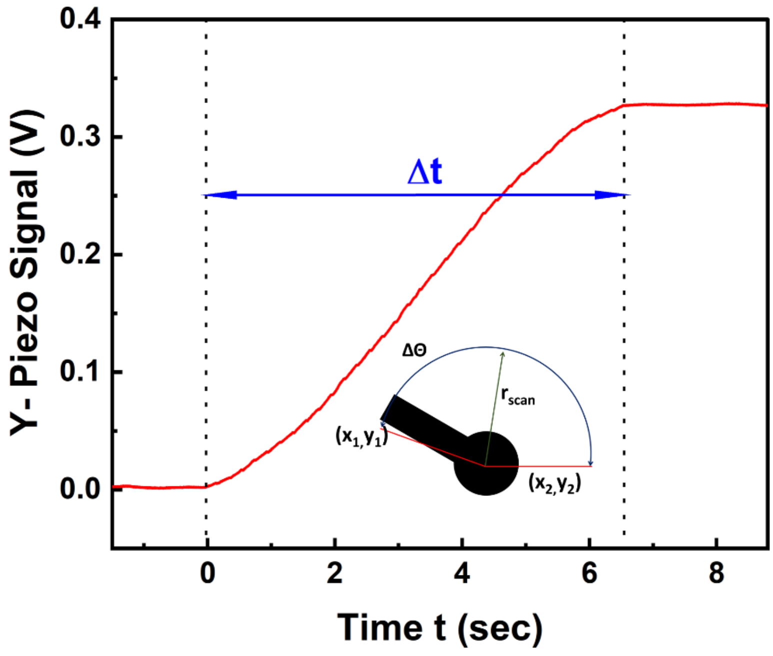

Figure S2. Time trace of cantilever displacemnt along the Y axis recorded from the oscilloscope. Blue arrow corresponds to the time duration for complete actuation.

4. Torque calculation

Torque is calculated as the product of F_{real} and the radius of the circular trajectory.

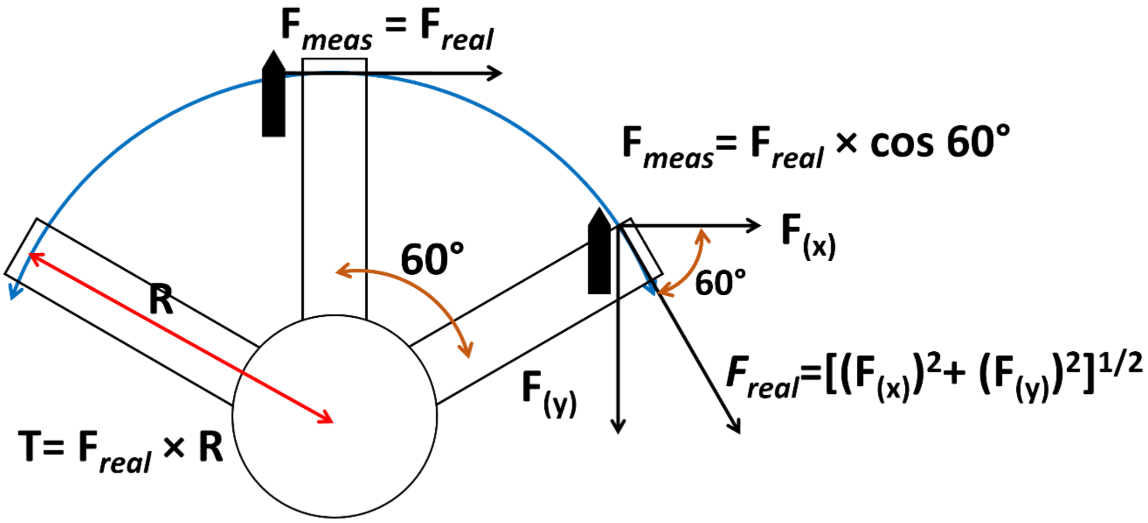

Figure S3. Force analysis of the interaction between AFM probe and lever arm during circular motion.

References

(1) Rokni, H.; Lu, W. Direct Measurements of Interfacial Adhesion in 2D Materials and van Der Waals Heterostructures in Ambient Air. *Nat. Commun.* **2020**, *11* (1). https://doi.org/10.1038/s41467-020-19411-7.

(2) Koren, E.; Rawlings, C.; Knoll, A. W.; Duerig, U. Adhesion and friction in mesoscopic graphite contacts. *Science* . **2015**, *348* (6235), 679–684.

(3) Bessler, R.; Duerig, U.; Koren, E. The Dielectric Constant of a Bilayer Graphene Interface. *Nanoscale Adv*. **2019**, *1* (5), 1702–1706. https://doi.org/10.1039/c8na00350e.

(4) Wang, W.; Dai, S.; Li, X.; Yang, J.; Srolovitz, D. J.; Zheng, Q. Measurement of the Cleavage Energy of Graphite. *Nat. Commun.* **2015**, *6*, 1–7. https://doi.org/10.1038/ncomms8853.
